# Integrated Proteomic and Metabolomic Analysis of an Artificial Microbial Community for Two-Step Production of Vitamin C

**DOI:** 10.1371/journal.pone.0026108

**Published:** 2011-10-07

**Authors:** Qian Ma, Jian Zhou, Weiwen Zhang, Xinxin Meng, Junwei Sun, Ying-jin Yuan

**Affiliations:** 1 Key Laboratory of Systems Bioengineering, Ministry of Education and Department of Pharmaceutical Engineering, School of Chemical Engineering and Technology, Tianjin University, Tianjin, People's Republic of China; 2 Welcome Pharmaceutical Co., Ltd. North China Pharmaceutical Group, Shijiazhuang, Hebei, People's Republic of China; The Scripps Research Institute, United States of America

## Abstract

An artificial microbial community consisted of *Ketogulonicigenium vulgare* and *Bacillus megaterium* has been used in industry to produce 2-keto-gulonic acid (2-KGA), the precursor of vitamin C. During the mix culture fermentation process, sporulation and cell lysis of *B. megaterium* can be observed. In order to investigate how these phenomena correlate with 2-KGA production, and to explore how two species interact with each other during the fermentation process, an integrated time-series proteomic and metabolomic analysis was applied to the system. The study quantitatively identified approximate 100 metabolites and 258 proteins. Principal Component Analysis of all the metabolites identified showed that glutamic acid, 5-oxo-proline, L-sorbose, 2-KGA, 2, 6-dipicolinic acid and tyrosine were potential biomarkers to distinguish the different time-series samples. Interestingly, most of these metabolites were closely correlated with the sporulation process of *B. megaterium*. Together with several sporulation-relevant proteins identified, the results pointed to the possibility that *Bacillus* sporulation process might be important part of the microbial interaction. After sporulation, cell lysis of *B. megaterium* was observed in the co-culture system. The proteomic results showed that proteins combating against intracellular reactive oxygen stress (ROS), and proteins involved in pentose phosphate pathway, L-sorbose pathway, tricarboxylic acid cycle and amino acids metabolism were up-regulated when the cell lysis of *B. megaterium* occurred. The cell lysis might supply purine substrates needed for *K. vulgare* growth. These discoveries showed *B. megaterium* provided key elements necessary for *K. vulgare* to grow better and produce more 2-KGA. The study represents the first attempt to decipher 2-KGA-producing microbial communities using quantitative systems biology analysis.

## Introduction

Microbial communities play essential roles in nature, making the most contributions to the earth's species diversity and being of great importance to various environmental, medical, and biotechnological applications [1], [2]. Intricate interactions between microbes are ubiquitous in various microbial communities ranging from bioreactors dominated by two species to more complex ecosystems, such as symbiotic human gut flora and sewage micro biota [3]. Much research in the past decades has focused on characterizing compositions and functionality of various microbial communities. Towards these goals, many technological methods have been developed and applied, including 16s-rRNA sequencing for community composition analysis [4], [5]; stable isotope probing technologies for linking microbial community function to phylogeny [6]; meta-genomics and meta-transcriptomics for capturing global view of genetic diversity and gene expression of communities [7]. A new approach, termed “meta-proteomics”, coined by Wilmes and Bond [8], identifies new functional genes, metabolic pathways and important proteins in response to environmental changes [9]. In recent years, a new research field, metabolomics which measures intracellular metabolites, advanced rapidly, with the potentials to bridge the gap between genetype and phenotype [10].

Although each of these systems biology platforms is important for the research of microbial communities, it is their use in combination that brings the most exciting potential [11]. Attempts to integrate various systems biology tools have been made in the study of single organisms. For example, integration of metabolomics, transcriptomics and fluxomics profiling in *Corynebacterium glutamicum* ATCC 13287 was performed at different stages of batch culture [12]. 2D-DIGE based proteomics and microarray based transcriptomics approaches were integrated in the study of adaptation of *Bacillus subtilis* to low temperature [13]. As another example, the correlation of transcriptome, proteome and metabolome for *Pyrococcus furiosus* undergoing a cold adaptation response had been performed to catalogue and correlate the overall molecular changes [14]. However, so far there are very few studies integrating data collected from different systems biology approaches for complex microbial communities.

In this study, we performed an integrated proteomic and metabolomic analysis of a simple artificial community consisting of two species, *Ketogulonicigenium vulgare* and *Bacillus megaterium*. Although other bacillus strains such as *Bacillus cereus*, *Bacillus thuringiensis* and *Bacillus macerans* could partly take the place of *B. megaterium* [15], it is the *B. megaterium* and *K. vulgare* community with excellent production ability and stability that has been constructed and widely used in industry for the production of 2-KGA, the precursor of vitamin C [16], [17], [18]. In our study, although adding purines to the *K. vulgare* mono-culture did have certain positive effect on the yields of 2-KGA, considering the high price of purines and the limited effect on the yield, it is the co-culture system that is more economic and efficient for industrial manufacture. It has been well documented that that the co-culture system has a higher efficiency of 2-KGA production, but it remains unclear how the two species interact and communicate with each other to achieve the production advantage during the industrial fermentation process. In our lab, the metabolic cooperation in the co-culture system was investigated by cultivating them spatially on a soft agar plate, and our results revealed the interactions between *B. megaterium* and *K. vulgare* were a synergistic combination of mutualism and antagonism [19]. In this study, with the goals to obtain molecular insights into this industry-important artificial microbial community and build a fundamental knowledge for engineering the community for improved production, we applied 2-dimensional electrophoresis (2-DE) along with high-resolution Gas Chromatography-Time of Flight-Mass Spectrometry (GC-TOF-MS) to obtain the proteomic and metabolomic profiles and variation patterns in the microbial community. The results showed that *Bacillus* sporulation process could be important for the interaction, in which the sporulation of *B. megaterium* could enhance the capability of *K. vulgare* to defend against oxidation stress. In addition, *Bacillus* lysis could supply more purine substrates for the enhanced growth and metabolism of *K. vulgare*.

## Results

### Growth of *K. vulgare* and *B. megaterium* and 2-KGA Production

**Figure 1:**
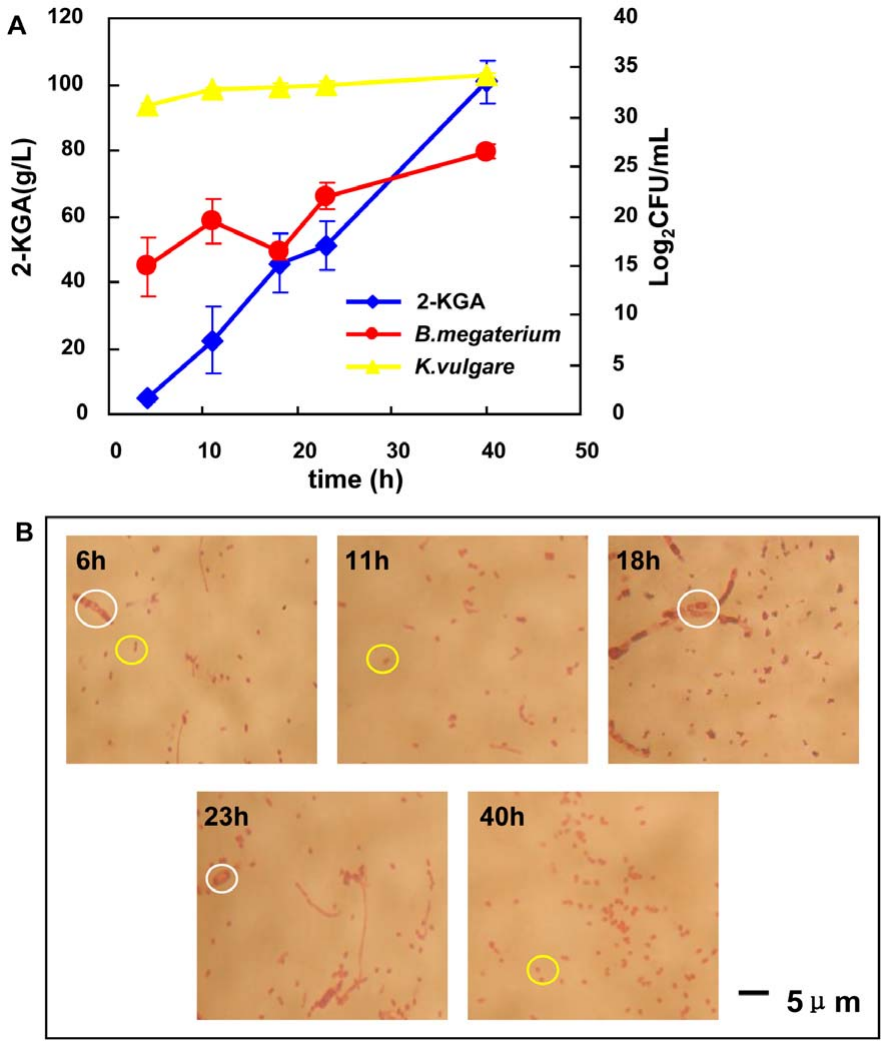
Growth, 2-KGA production and bacteria morphology status of the consortium: (A) growth curves of *K. vulgare* and *B. megaterium* and 2-KGA production; (B) microscopic observation of the consortium at the sampling points of 6 h, 11 h, 18 h, 23 h and 40 h. *B. megaterium* spores were indicted by white circles, and *K. vulgare* cells were indicted by yellow circles.

Based on the different colony morphologies for *K. vulgare* and *B. megaterium*, cell growths rates of these two species were evaluated by counting their colony forming units (CFUs) one milliliter of fermentation broth. Cells were harvested at 6 h, 11 h, 18 h, 23 h and 40 h of the fermentation process (Fig. 1 A). After the initial lag phase, both *K. vulgare* and *B. megaterium* grew fast from 6 h to 11 h. From the microscopic examinations shown in Fig. 1 B, *B. megaterium* sporulation became visible at 6 h, which was probably due to the 4°C storage of seed liquor. More significant sporulation of *B. megaterium* occurred at 18 h. Later on, cell lysis occurred at 23h and few visible spores existed. Additionally, by microscopy observation and cell counting, we calculated the percentage of sporulation of *B. megaterium* cells at 6 h, 11 h, 18 h, 23 h and 40 h were 10%, 0%, 90%, 90%, and 40%, respectively. The concentration of 2-KGA was determined by High Performance Liquid Chromatography (HPLC), as shown in Fig. 1 A. Additional L-sorbose liquor from the first step fermentation was added at 19 h, causing the slight decrease of 2-KGA concentration at 23 h as a result of the dilution. After the supplement of L-sorbose liquor, two bacteria went through an additional growth and the final concentration of 2-KGA reached 100.9 g/L at the end of the fermentation.

### Proteomic profiling for the consortium

**Figure 2:**
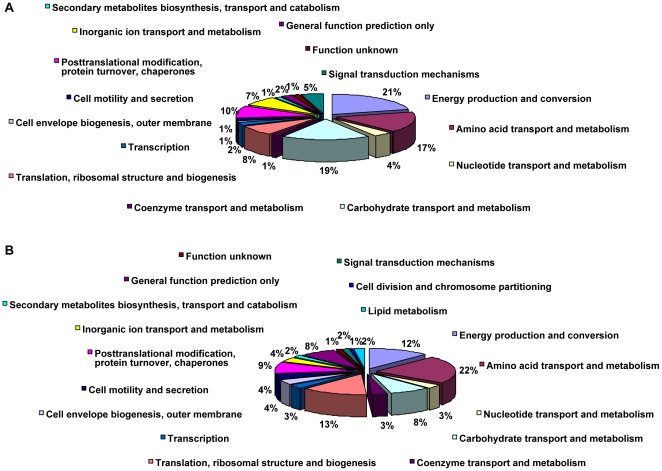
Protein function classifications of *B. megaterium* and *K. vulgare*: (A) function classification of *B. megaterium*; (B) function classification of *K. vulgare*. The classification was made by searching the database of Clusters of Orthologous Groups of proteins using Cognitor. Relative proportions of each ontology term were expressed in percentage values.

A total of 258 proteins were identified by MALDI-TOF/TOF-MS and quantified by 2-D gel image analysis, as shown in Table S1. Among them, 179 proteins belonged to *K. vulgare*, and 79 belonged to *B. megaterium*. To get an overview of identified protein functions, phylogenetic classification of proteins was performed separately for each species by searching the database of Clusters of Orthologous Groups of proteins (http://www.ncbi.nlm.nih.gov/COG) by COGnitor. As shown in Fig. 2, proteins for energy production and conversion, followed by carbohydrate transport and metabolism, and amino acid transport and metabolism predominated in *B. megaterium*. While in *K. vulgare*, proteins involved in amino acids transport and metabolism; translation, ribosomal structure and biogenesis, energy production and conversion were among the greatest number of proteins synthesized categories.

**Figure 3:**
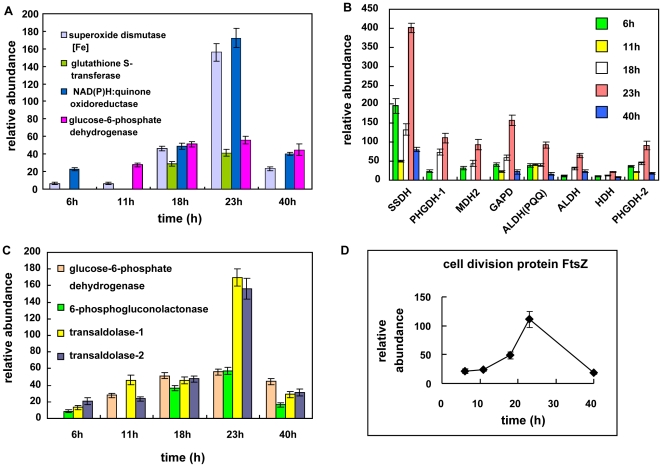
Profiles of identified proteins in *K. vulgare* against ROS and participating in important metabolic pathways: (A) proteins against ROS; (B) proteins participating in the PPP pathway; (C) proteins involved in sorbose pathway, TCA cycle and other important processes (SSDH: sorbose/sorbosone dehydrogenase; PHGDH: phosphoglycerate dehydrogenase; MDH: malate dehydrogenase; GAPD: glyceraldehyde-3-phosphate dehydrogenase; ALDH (PQQ): membrane-bound aldehyde dehydrogenase [pyrroloquinoline-quinone]; ALDH: aldehyde dehydrogenase family protein; HDH: homoserine dehydrogenase). (D) relative abundance of protein FtsZ.

In our study two groups of enzymes involved in antioxidant protection were comprehensively investigated in *K. vulgare*. One is superoxide dismutase which metabolizes superoxide anion for cells against ROS, and the others are proteins including glutathione S-transferase, NADPH: quinone oxidoreductase and glucose-6-phosphate dehydrogenase, all of which involve glutathione for the detoxication of ROS-modified compounds. These two mechanisms work together to maintain a reducing environment for cellular metabolism. The expression levels of key proteins involved in these two mechanisms were determined and depicted in Fig. 3 A, and the results showed that they reached a higher level of expression at 18–23 h for the microbial consortium.

Several important dehydrogenases in *K. vulgare* were also found up-regulated, shown in Fig. 3 B. They were enzymes involved in L-sorbose pathway (sorbose/sorbosone dehydrogenase), glycolytic pathway (glyceraldehyde-3-P dehydrogenase), TCA (malate dehydrogenase) and amino acids metabolism (D-3-phosphoglycerate dehydrogenase, homoserine dehydrogenase). It was worth noting that expression levels of these proteins at 6 h were higher than other sampling points other than 18 h and 23 h, coincided with the sporulation of *B. megaterium* at 6 h, 18 h and 23 h.

To find out how *K. vulgare* was benefiting from its partner in the consortium, we examined its carbohydrate metabolism pathway. Pentose Phosphate Pathway (PPP) was reported to be the central carbohydrate metabolism pathway in *K. vulgare* [20]. In our study, three enzymes were identified for this pathway including 6-phosphogluconolactonase, glucose-6-phosphate dehydrogenase and transaldolase, with glucose-6-phosphate dehydrogenase being the rate-controlling enzyme. As shown in Fig. 3 C, when the sporulation of *B. megaterium* came to a maximum extent, the expressions of these enzymes at 18 h and 23 h were more than two folds up-regulated compared with that at 6 h. The proliferation of *K. vulgare* was promoted by the addition of the second bacteria *B. megaterium*, supported by the increased expression of cell division protein FtsZ, as shown in Fig 3 D. Expression of FtsZ was up-regulated about 2-folds at 18 h and 4-folds at 23 h, respectively. Several spots of subunits of ATP synthases in *K. vulgare* were identified on the gel, suggesting of post-translational modifications, and the expression of these synthases were higher at 18 h and 23 h, indicating the energy production of *K. vulgare* was accelerated.

### Metabolomic Profiling for the Consortium

**Figure 4:**
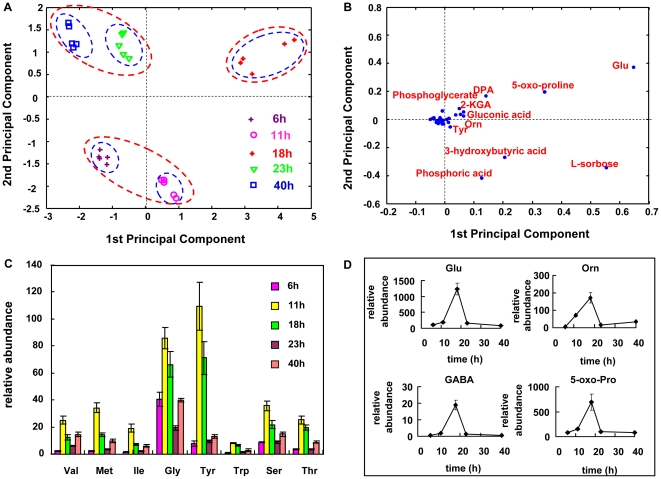
Principal Component Analysis of metabolomic data and profiles of some important amino acids: (A) scores plot of the time series samples; (B) loading plot of PC1 vs. PC2; (C) amino acids with the similar pattern: contents at 11 h were higher than other sampling time; (D) amino acids as potential biomarkers.

GC-TOF-MS was applied to detect the intracellular metabolites of the co-cultivated bacteria during the fermentation process. A total of 100 metabolites were identified and quantified, including amino acids, sugars, organic acids, amines, alcohols and their derivatives. Principal Component Analysis (PCA) was carried out using Matlab 7.0 to find the possible biomarkers responsible for different status of the consortium during fermentation. As shown in Fig. 4 A, the samples from different fermentation time points can be distinguished clearly in the scores plot. In addition, the early, middle and late stages of the fermentation can be distinguished, as indicated by the red circles in Fig. 4 A. In order to further analyze which metabolites contribute the most to this discrimination, loadings plot analysis was performed, as shown in Fig. 4 B. Glutamic acid, 5-oxo-proline, L-sorbose, 2-KGA, phosphoric acid, 3-hydroxybutyric acid, 2, 6-dipicolinic acid, gluconic acid, tyrosine and ornithine were identified as the potential biomarkers to distinguish different fermentation stages.

The profiles of amino acids were shown in Fig. 4 C. Eight amino acids had a similar change trend: increased fast from 6 h to 11 h, and climbed to a relative maximum from 11 h to 18 h, then decreased accompanying the sporulation initiation of *B. megaterium* from 18 h to 23 h, and after the addition of L-sorbose liquor, a slight increase was observed from 23 h to 40 h. Fig. 4 D showed amino acids with similar pattern of changes across fermentation process as 2, 6-dipicolinic acid, a chemical compound unique to the *Bacillus* genus and when coupled to calcium would account for the heat resistance of spores, these results indicated that sporulation was an important event in the consortium and had a close correlation with amino acids metabolism.

### Correlations between Proteins and Metabolites

**Figure 5:**
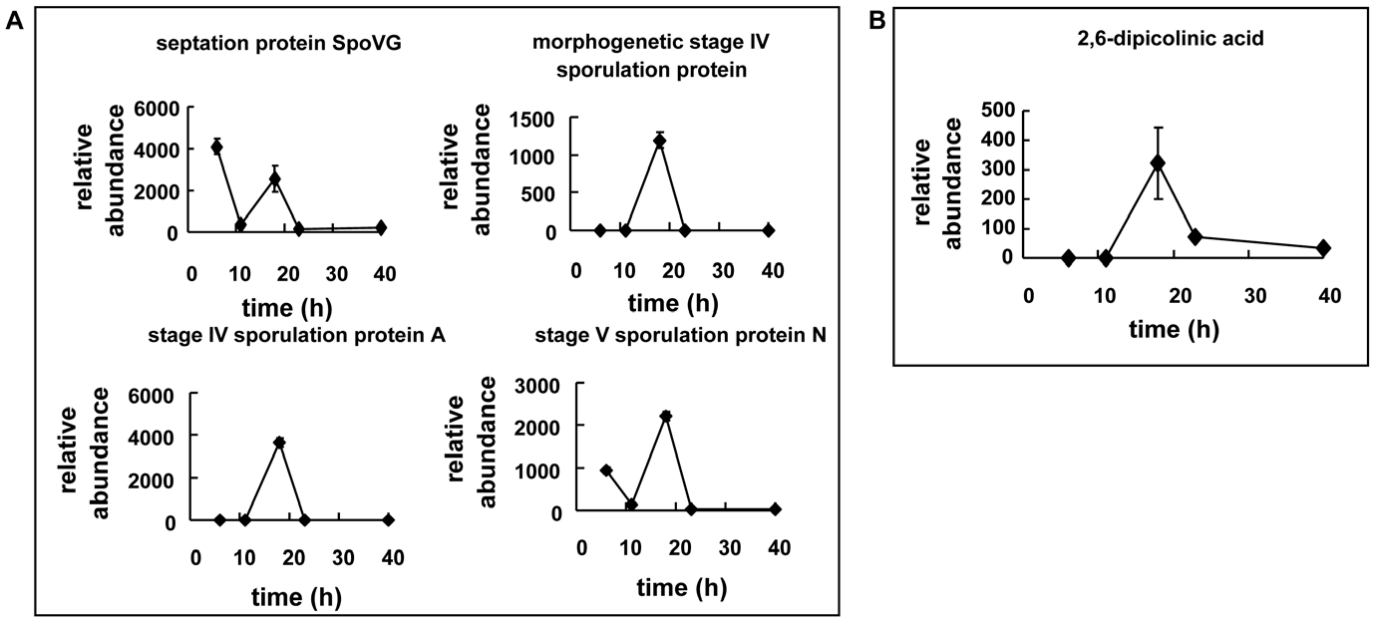
Correlations between proteins and metabolites: (A) sporulation proteins of: the relative expression level of four sporulation proteins in time series; (B) relative intracellular content of 2, 6-dipicolinic acid in *B. megaterium*.

Four sporulation proteins were identified and their relative expression levels were depicted in Fig. 5A. Septation protein SpoVG, participating in the septum forming within the mother cell, expressed from the onset of sporulation, evidenced by the microscope observation at 6 h in Fig. 1 B. Stage IV sporulation protein A was only detected at 18 h, and the expression levels of morphogenetic stage IV sporulation protein and stage V sporulation protein reached the highest at 18 h, consistent with the intracellular 2, 6-dipicolinic acid content, as shown in Fig. 5 B. Together they indicated that sporulation progressed to a great extent at 18 h, evidenced in Fig. 1 B. Moreover, these sporulation proteins were not detected at 23 h, suggesting the lysis of cells occurred at that time.

**Figure 6:**
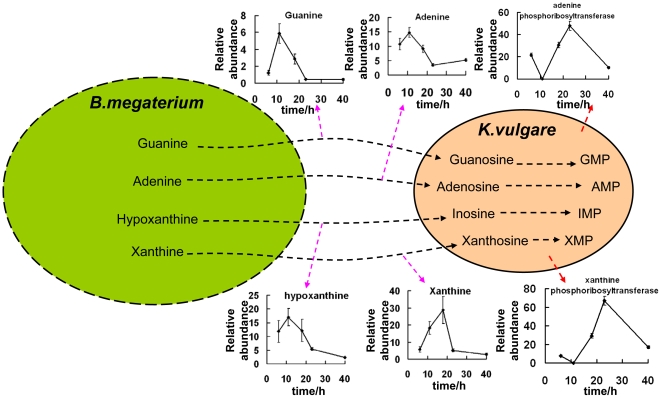
Purine flow between *K. vulgare* and *B. megaterium*: X-axis denoted time with a unit of hour; Y-axis denoted relative abundance of metabolites or proteins.

**Figure 7:**
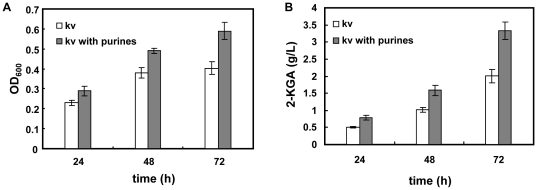
Growth and 2-KGA production of *K. vulgare* with and without purines (adenine, guanine, xanthine, hypoxanthine, 0.05g/L) addition: (A) Optical density (600nm) of *K. vulgare* at 24 h, 48 h and 72 h of fermentation; (B) 2-KGA production of *K. vulgare* at 24 h, 48 h and 72 h of fermentation, and “kv” represents *K. vulgare*.

After 18h, the relative abundance of intracellular guanine, adenine, hypoxanthine and xanthine in the consortium declined as shown in Fig. 6, while the relative expression levels of adenine phosphoribosyltransferase and xanthine phosphoribosyltranserase in *K. vulgare* had a sharp increase at 18 h and 23 h. A mixture of guanine, adenine, hypoxanthine and xanthine (Sigma-Aldrich), with each component having a final concentration of 0.05g/L, was added to *K. vugare* mono-culture. The growth and 2-KGA production status at 24 h, 48 h, and 72 h of fermentation were shown in Fig. 7. The purine mixture had improved the growth of *K. vulgare* to about 1.4 folds, and 2-KGA production to about 1.6 folds.

## Discussion

In industry fermentation, two species *K. vulgare* and *B. megaterium* were cultivated together to generate 2-KGA. During the process, we observed maximum sporulation of *B. megaterium* at 18 h and its cell lysis later at 23 h. To understand the correlations between these phenomena and 2-KGA production, we performed an integrated proteomic and metabolomic analysis of the artificial consortium for industrial VC production using 2-DE coupled MALDI-TOF/TOF-MS and GC-TOF-MS approaches. Altogether from several samples collected through the fermentation process, we identified 258 proteins and 100 metabolites. The analysis and interpretation of the quantification data showed that *B. megaterium* helped *K. vulgare* growth probably by providing a better living environment and supplying essential substances such as substrates for purine nucleotides synthesis.

Among the potential biomarkers found in PCA loadings plot, glutamic acid contributed the most to distinguish the 18 h sample characterized by maximum sporulation of *B. megaterium*. Glutamic acid has been reported to serve as an amino donor in sporulating cells, precursor to 2, 6-dipicolinic acid biosynthesis and also as a carbon and nitrogen substrate for the synthesis of other amino acids during the transition of a vegetative cell to a spore [21]. 5-oxo-proline accounts for the second largest contribution to distinguish the sporulation stage, formed from γ-glutamyl amino acids by the γ-glutamylcyclotransferase [22]. 5-oxo-proline can be converted to glutamic acid by 5-oxo-L-proline amidohydrolase in the glutathione metabolism pathway [23]. Detection of these biomarkers in the consortium pointed to the possibility that *B. megaterium* sporulation had occurred, which was evidenced by microscopic observation as well. From this point of view, these metabolites identified could be potential biomarkers for the sporulation process of *B. megaterium*.

During the sporulation of *B. megaterium*, proteins related to sporulation function are synthesized [24]. The septation protein SpoVG that regulates asymmetric septation during the second stage of sporulation is synthesized early in the stationary phase in *B. megaterium* [25]. In our study, the septation protein SpoVG and stage V sporulation protein N were both synthesized at 6 h, suggesting that a heterogeneous population of *B. megaterium* in which cells were in different stages of sporulation might originated from the cooled seed liquor. 2, 6-dipicolinic acid was found almost exclusively in bacterial endospores with high concentrations, which had been reported to be responsible for the resistance of spores when combined with calcium [26]. Thus, the existence of 2, 6-dipicolinic acid could be used to infer the sporulation stage of *B. megaterium*. These clues in protein synthesized correlated well with respective sporulation-related metabolites. This correlation of proteins and metabolites also suggested the significance of sporulation to the consortium.

ROS like superoxide anion, hydrogen peroxide, and hydroxyl radical are the results of oxygen metabolism in aerobic organisms [27]. ROS can attack DNA, resulting in chain breaks, modification of the carbohydrate parts and nitro bases, and may lead to point mutation [28]. ROS can also modify amino acids in proteins [27]. During the ROS defense processes, NADPH provides the reducing power provided by glucose-6-phosphate dehydrogenase [27], [29]. If one microbial organism doesn't have enough reducing power, the detoxication of radicals by antioxidant enzymes will be limited, and thus the growth and metabolism of the organism will be impaired. Due to the poor PPP metabolism of *K. vulgare*, the production of NADPH may be insufficient. After the sporulation of *B. megaterium*, the enzyme for NADPH production was up-regulated, and at the same time, enzymes for the detoxication of radicals were also up-regulated. It has been reported that the infection of *B. popilliae* to larvae caused the oxidation-reduction potential decrease in larvae blood [30]. Heavy inoculum of *B. megaterium* was also reported to shift the potential of the medium to the negative side [31]. It is possible that *B. megaterium* may also behave the similar way in this consortium. The results point to the possibility that the sporulaton process of *B. megaterium* could help *K. vulgare* in its combat against ROS. Together with the help of *B. megaterium* in central carbohydrate metabolism pathway and ATP synthases, *K. vulgare* acquired a better survival capability.

Leduc et al. (2004) studied the growth factor requirements of *K. vulgare* LMP P-20356 under L-sorbose/CSL medium [32], and the results showed that adenine, guanine, thymine and folates could enhance its growth significantly, suggesting that the purine nucleotides and desoxythymidylate biosynthesis pathways in *K. vulgare* were probably insufficient. In response to starvation of carbon, nitrogen or phosphorous sources [26], *B. megaterium* can initiate formation of septum that asymmetrically divides the cell into a larger mother cell and a smaller forespore [33], [34], and then after a series of sporulation stages, the mother cell engulfs the future daughter cell, and eventually actively lyses prior to spore release [35]. With the lysis of mother cell, peptides, proteins, purines, pyrimidines and small molecules in its cytoplasm can be released. In our study, the adenine, guanine, xanthine and hypoxanthine were likely from the lysis of *B. megaterium* after 18 h. At the same time, up-regulation of xanthine phosphoribosyltransferase and adenine phosphoribosyltransferase in *K. vulgare* were also observed, suggesting that the purine pathway in *K. vulgare* was accelerated. In our experiment, the addition of purine mixture showed improved growth and 2-KGA production of *K. vulgare*, consistent with previous research [32], showing the purine demand of *K. vulgare*. Thus it was speculated that the sporulation and cell lysis of *B. megaterium* provided substrates for the nucleotides synthesis of *K. vulgare*.

In summary, we used integrated proteomics and metabolomics to study an artificial microbial community consisted of *K. vulgare* and *B. megaterium*. The quantification data provides new insights to how the microbes interact with each in the community. The results demonstrated that *B. megaterium* could help *K. vulgare* to resist ROS, enhance its energy producing and promote its growth and metabolism also by providing essential components such as purine during sporulation.

## Materials and Methods

### Strains and Industrial Fermentation Conditions

The two industrial strains, *K. vulgare* and *B. megaterium*, used in this study for vitamin C fermentation were provided by Welcome Pharmaceutical Co., Ltd. North China Pharmaceutical Group (Shijiazhuang, Hebei, China). The seed medium contains: 20 g/L L-sorbose, 1 g/L KH_2_PO_4_, 3 g/L yeast powder, 0.2 g/L MgSO_4_, 3 g/L beef extract, 1 g/L urea, 10 g/L peptone, and 3 g/L corn steep liquor. The fermentation medium contains: 80 g/L L-sorbose (fed at the beginning and the middle phases of the fermentation), 20 g/L corn steep liquor, 1 g/L KH_2_PO_4_, 0.2 g/L MgSO_4_, and 12 g/L urea. The fermentation volume is 240,000 L with a 10% inoculum. The pH values of seed and fermentation media were maintained at 6.8 and 7.0 respectively by the addition of Na_2_CO_3_. The temperature was 30°C, and fermentation typically lasted for about 40 h.

### Analysis of L-sorbose and 2-KGA in Fermentation Broth

L-sorbose and 2-KGA in the fermentation broth were analyzed by HPLC (Waters Corp., Massachusetts, USA) with a refractive index detector. 5 mM H_2_SO_4_ was used as the eluent on an Aminex HPX-87H column (BioRad, CA) at the temperature of 65°C with a flow rate of 0.6 mL/min.

### Determination of the Approximate Number of Two Bacteria

1.0 mL fermentation broth was diluted into several gradients with sterilized water, and then 100 µL of each gradient was spread on agar plate respectively. The Colony Forming Units (CFUs) of *B. megaterium* and *K. vulgare* in 1.0 mL broth could be calculated thanks to the colony morphology differences of these two bacteria, with colonies of *K. vulgare* being transparent and having diameters of about less than 0.5 millimeter, while colonies of *B. megaterium* being white and having diameters of more than 3 millimeters.

### Sampling and Quenching

At 6 h, 11 h, 18 h (one hour before the L-sorbose addition), 23 h and 40 h of the fermentation, cells were harvested by a centrifugation at 500×g for 3 min at 4°C to precipitate the solid ingredients in the medium followed by another centrifugation at 8000 ×g for 10 min at 4°C. The supernatants were collected for 2-KGA analysis, while the pellets were quenched by liquid nitrogen after washing twice with phosphate buffer solution (PBS, pH 7.2) and once with dd-H_2_O. Then the pellets were stored at −40°C until use.

### Extraction, Derivation and Analyses of Metabolites

Extraction of metabolites was performed according to the method described by Ding et al. [36] with minor modifications. Briefly, 50 mg cells were ground in liquid nitrogen with mortar and pestle, and then were suspended in 1 mL of extraction buffer containing methanol/water (1∶1, v/v, −40°C), vortexed to mix, frozen and thawed three times to further disrupt the cells. After centrifugation at 5000×g for 5 min at 4°C, 150 µL supernatant of each sample was transferred to a new tube. Succinic d_4_ acid solution (0.14 mg/mL, 50 µL) was added to the tube as an internal standard to correct possible analytical variations. Next, the mixture was vortexed, precipitated and lyophilized. For each sample, five replicates were performed.

Subsequent two-stage chemical derivatization was performed as described [37]: methoxamine hydrochloride (20 mg/mL in pyridine, 50 µL) and N-methyl-N-(trimethylsilyl) trifluoroacetamide (MSTFA, 80 µL) were sequentially added to the dry samples and incubated at 30°C for 90 min and 37°C for 30 min respectively, to achieve the methoximation of carbonyl group and the trimethylsilylation of reactive hydrogen.

GC-TOF-MS analysis of metabolites was performed as described before with minor modifications [36]. A total of 1 µL sample was injected into the GC (Agilent 6890) equipped with a fused-silica DB-5 capillary column (30 m×0.25 mm i.d., 0.25 µm, J&W Scientific, Folsom, CA). The injector and ion source temperatures were 280°C and 250°C respectively, with the oven temperature set as the following: 70°C for 2 min, increased to 290°C for 3 min with a slope of 8°C /min. 70 eV electron beam and 40 µA ionization current were applied to generate appropriate ions. The spectrum acquisition range was set to 50–800 m/z.

Masslynx software (Version 4.1, Waters Corp., USA) was used for mass peak identification and quantification. Metabolites identification was carried out by automatically searching the National Institute of Standards and Technology mass spectral library (NIST 2005), supplemented with manual corrections. The quantification of metabolites was conducted by calculating the areas under respective peaks after normalization against the internal standard. After mean centering, the data of metabolites abundances were subjected to Principal Component Analysis (PCA) by Matlab 7.0.

### Extraction, 2-DE, Image Analysis and Identification of Intracellular Proteins

Extraction of intracellular proteins was performed according to the method by Cheng et al. [38]. First, 0.2 g cells ground in liquid nitrogen with mortar and pestle were suspended in 1 mL of cell lysis buffer (7 M urea, 2 M thiourea, 4% m/v CHAPS, 40 mM Tris-HCl, and 1 mM PMSF) at 4°C for 2 h. After centrifugation at 12000 xg for 40 min at 4°C, the supernatant was transferred to another tube, and 5 times volume of ice-cold acetone was added to precipitate the proteins at −40°C overnight. After extraction, protein content was determined using the Bradford method [39]. Then, approximate 800 µg proteins in 350 µL rehydration buffer were loaded on the immobilized pH gradient (IPG) dry strips (18 cm long, pH 4–11, GE Healthcare, UK). The isoelectric focusing (IEF) was carried out under the following voltage program: 30 V for 12–14 h, 500 V for 1 h, and 8000 V for 8 h. Following the subsequent equilibration in SDS equilibration buffer, 12.5% SDS-PAGE was conducted at 15°C for about 20 h until the bromophenol blue reached the bottom of each gel. Each experiment was carried out at least twice for replicates. 2-DE analysis was performed as described before [38]. Coomassie blue R250 was used to stain the gels, and subsequently, gels were scanned by an Image Scanner (Amersham Biosciences, Uppsala, Sweden). ImageMaster 2-D Elite version 3.01 (Amersham Biosciences) was used for quantitative imaging analysis of protein spots to obtain their relative abundances. The protein spots of interest were excised from gels, washed by dd-H_2_O, destained by acetonitrile (50%) and 25 mM (NH_4_)HCO_3_, dehydrated by acetonitrile (100%), dried by Speed-Vac system and in-gel digested by trypsin at 37°C overnight. MALDI-TOF MS was performed using Autoflex TOF-TOF II (Bruker Daltonics, Germany). After the peptide mass fingerprint of each protein was obtained, the LIFT mode was chosen to perform the MS/MS analysis, resulting in the MS/MS spectrum of each selected peptide. MASCOT (http://www.matrixscience.com) was applied to identify proteins by searching an in-house database constructed by combining the protein sequences of *K. vulgare* we sequenced and those of *Bacillus* genus in the National Center for Biotechnology Information database. The parameters used to do the searching were: maximum missed cleavages set to 1; peptide mass tolerance set to ±100 ppm and fragment mass tolerance set to ±0.5 Da. We required a matching of two or more peptides per protein as a confident detection. InterPro (http://www.ebi.ac.uk/interpro/) was used for protein sequence analyses and classification, and the Kyoto Encyclopedia of Genes and Genomes (http://www.genome.jp/kegg/pathway.html) was used to reconstruct major metabolic pathways in two microbes. Database of Clusters of Orthologous Groups of proteins (http://www.ncbi.nlm.nih.gov/COG) was utilized to perform phylogenetic classification of proteins [40], [41].

### Data normalization

Total proteins of the two strains were extracted without distinguishing the source. As the ratio of two bacteria varied during the fermentation process, normalization of the obtained proteomics data was needed. The method for normalization is illustrated as below: taking *K. vulgare* as an example, let a1, a2, a3, a4, a5 denote the relative protein expression values obtained after quantification for 6 h, 11 h, 18 h, 23 h and 40 h, respectively, and the corresponding numbers for the sampling points are n1, n2, n3, n4, n5 for *K. vulgare* and n11, n22, n33, n44, n55 for *B. megaterium*. The mass of per *K. vulgare* and *B. megaterium* are m1 and m2, respectively. The normalized protein expression values can be calculated according to the following equation:
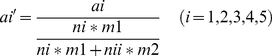
(1)m1 and m2 were obtained by culturing the two bacteria separately on agar plates, collecting the cells using sterile water, calculating the CFUs, weighing the total mass, and then dividing the total mass by CFUs. The normalization for *B. megaterium* was performed using the similar approach.

## Supporting Information

Table S1 Proteins identified by MALDI-TOF/TOF-MS for the consortium.
(DOC)
