# Supplementary material for: Integrated Proteomic and Metabolomic Analysis of an Artificial Microbial Community for Two-Step Production of Vitamin C
Source: PLoS One. 2011 Oct 7;6(10):e26108. doi: 10.1371/journal.pone.0026108 (PMC3189245; doi:10.1371/journal.pone.0026108)
Supplement: Table S1 — Proteins identified by MALDI-TOF/TOF-MS for the consortium. (DOC) [file pone.0026108.s001.doc]

**Table S1.** **Proteins identified by MALDI-TOF/TOF-MS for the consortium**

| Protein name | accession | Nominal Mr | Calculated PI | species | protein score(p<0.05) |
| --- | --- | --- | --- | --- | --- |
| DNA polymerase III, beta subunit | gkv_981 | 40013 | 4.89 | *Ketogulonicigenium vulgare* | 271 |
| ahpC/TSA family protein | gkv_979 | 23749 | 5.16 | *Ketogulonicigenium vulgare* | 182 |
| aldehyde dehydrogenase family protein | gkv_977 | 48875 | 5.12 | *Ketogulonicigenium vulgare* | 164 |
| bifunctional purine biosynthesis protein PurH | gkv_971 | 55494 | 5.03 | *Ketogulonicigenium vulgare* | 312 |
| insulinase (Peptidase family M16) family protein | gkv_967 | 47401 | 4.56 | *Ketogulonicigenium vulgare* | 92 |
| bacterial extracellular solute-binding proteins, family 5 Middle family protein | gkv_905 | 55063 | 4.65 | *Ketogulonicigenium vulgare* | 355 |
| bacterial extracellular solute-binding proteins, family 5 Middle family protein | gkv_905 | 55063 | 4.65 | *Ketogulonicigenium vulgare* | 293 |
| bacterial extracellular solute-binding proteins, family 5 Middle family protein | gkv_905 | 55063 | 4.65 | *Ketogulonicigenium vulgare* | 280 |
| metE | gkv_900 | 38408 | 5.34 | *Ketogulonicigenium vulgare* | 220 |
| metE | gkv_900 | 38408 | 5.34 | *Ketogulonicigenium vulgare* | 35 |
| arginyl-tRNA synthetase | gkv_861 | 63606 | 5.34 | *Ketogulonicigenium vulgare* | 53 |
| inositol-1-monophosphatase (IMPase) (Inositol-1-phosphatase) (I-1-Pase) | gkv_807 | 29677 | 5.52 | *Ketogulonicigenium vulgare* | 40 |
| acetylornithine/succinyldiaminopimelate aminotransferase (ACOAT) (Succinyldiaminopimelate transferase)(DapATase) | gkv_788 | 41439 | 5.12 | *Ketogulonicigenium vulgare* | 180 |
| ornithine carbamoyltransferase | gkv_787 | 34087 | 5.73 | *Ketogulonicigenium vulgare* | 96 |
| serine hydroxymethyltransferase (Serine methylase)(SHMT) | gkv_776 | 46126 | 5.79 | *Ketogulonicigenium vulgare* | 177 |
| tol-Pal system beta propeller repeat protein TolB | gkv_740 | 47353 | 6.15 | *Ketogulonicigenium vulgare* | 277 |
| glyceraldehyde-3-phosphate dehydrogenase, type I | gkv_718 | 35480 | 5.57 | *Ketogulonicigenium vulgare* | 188 |
| glutamate racemase | gkv_708 | 29318 | 5.51 | *Ketogulonicigenium vulgare* | 84 |
| citrate (Si)-synthase | gkv_703 | 48491 | 5.76 | *Ketogulonicigenium vulgare* | 101 |
| basic membrane family protein | gkv_7 | 38520 | 4.55 | *Ketogulonicigenium vulgare* | 168 |
| phosphopyruvate hydratase | gkv_699 | 45503 | 4.66 | *Ketogulonicigenium vulgare* | 234 |
| phosphopyruvate hydratase | gkv_699 | 45503 | 4.66 | *Ketogulonicigenium vulgare* | 188 |
| bacterial extracellular solute-binding family protein | gkv_682 | 45060 | 4.38 | *Ketogulonicigenium vulgare* | 251 |
| glycyl-tRNA synthetase, beta subunit | gkv_655 | 74152 | 5.17 | *Ketogulonicigenium vulgare* | 163 |
| cell division protein FtsZ | gkv_514 | 57491 | 5.02 | *Ketogulonicigenium vulgare* | 96 |
| lipase | gkv_492 | 33165 | 5.37 | *Ketogulonicigenium vulgare* | 130 |
| sorbose/sorbosone dehydrogenase | gkv_362 | 60624 | 4.39 | *Ketogulonicigenium vulgare* | 183 |
| bacterial extracellular solute-binding proteins, family 5 Middle family protein | gkv_3186 | 59309 | 4.49 | *Ketogulonicigenium vulgare* | 222 |
| bacterial extracellular solute-binding proteins, family 5 Middle family protein | gkv_3186 | 59309 | 4.49 | *Ketogulonicigenium vulgare* | 179 |
| dipeptidase, family protein | gkv_3183 | 54435 | 4.86 | *Ketogulonicigenium vulgare* | 105 |
| D-isomer specific 2-hydroxyacid dehydrogenase, NAD binding domain protein | gkv_3127 | 36712 | 5.16 | *Ketogulonicigenium vulgare* | 254 |
| hydantoinase/oxoprolinase family protein | gkv_3032 | 74910 | 5.05 | *Ketogulonicigenium vulgare* | 83 |
| transaldolase, putative | gkv_3003 | 23633 | 4.75 | *Ketogulonicigenium vulgare* | 74 |
| transaldolase, putative | gkv_3003 | 23633 | 4.75 | *Ketogulonicigenium vulgare* | 71 |
| conserved hypothetical protein | gkv_2991 | 13170 | 5.88 | *Ketogulonicigenium vulgare* | 128 |
| conserved hypothetical protein | gkv_2991 | 13170 | 5.88 | *Ketogulonicigenium vulgare* | 146 |
| clp protease family protein | gkv_2989 | 23110 | 5.5 | *Ketogulonicigenium vulgare* | 65 |
| acetyl-CoA carboxylase, biotin carboxylase | gkv_2984 | 48873 | 5.6 | *Ketogulonicigenium vulgare* | 174 |
| acetyl-CoA carboxylase, biotin carboxyl carrier protein | gkv_2983 | 16895 | 4.79 | *Ketogulonicigenium vulgare* | 108 |
| metallopeptidase family M24 family protein | gkv_2982 | 64986 | 5.21 | *Ketogulonicigenium vulgare* | 314 |
| NADPH-dependent FMN reductase family protein | gkv_2979 | 21663 | 5.09 | *Ketogulonicigenium vulgare* | 237 |
| cobaltochelatase, CobS subu | gkv_2978 | 36418 | 5.66 | *Ketogulonicigenium vulgare* | 109 |
| glutamyl-tRNA(Gln) and/or aspartyl-tRNA(Asn) amidotransferase, B subunit | gkv_2974 | 55174 | 4.98 | *Ketogulonicigenium vulgare* | 116 |
| bacterial extracellular solute-binding proteins, family 5 Middle family protein | gkv_2946 | 57986 | 4.55 | *Ketogulonicigenium vulgare* | 294 |
| cytosol aminopeptidase family, catalytic domain protein | gkv_2912 | 52062 | 5.97 | *Ketogulonicigenium vulgare* | 78 |
| glucose-6-phosphate dehydrogenase | gkv_2887 | 54060 | 5.45 | *Ketogulonicigenium vulgare* | 189 |
| 6-phosphogluconolactonase | gkv_2886 | 24054 | 5.13 | *Ketogulonicigenium vulgare* | 154 |
| hypothetical protein | gkv_2825 | 29657 | 5.29 | *Ketogulonicigenium vulgare* | 375 |
| hypothetical protein | gkv_2825 | 26174 | 5.27 | *Ketogulonicigenium vulgare* | 43 |
| bacterial extracellular solute-binding proteins, family 5 Middle family protein | gkv_2823 | 60022 | 4.65 | *Ketogulonicigenium vulgare* | 292 |
| bacterial extracellular solute-binding proteins, family 5 Middle family protein | gkv_2823 | 60022 | 4.65 | *Ketogulonicigenium vulgare* | 198 |
| luciferase-like monooxygenase family protein | gkv_2819 | 36264 | 5.88 | *Ketogulonicigenium vulgare* | 115 |
| luciferase-like monooxygenase family protein | gkv_2819 | 36264 | 5.88 | *Ketogulonicigenium vulgare* | 68 |
| uncharacterized peroxidase-related enzyme family protein | gkv_2817 | 22127 | 4.63 | *Ketogulonicigenium vulgare* | 297 |
| ribosome recycling factor | gkv_2802 | 20906 | 5.01 | *Ketogulonicigenium vulgare* | 61 |
| nitroreductase family protein | gkv_2740 | 29369 | 5.95 | *Ketogulonicigenium vulgare* | 65 |
| bacterial extracellular solute-binding proteins, family 5 Middle family protein | gkv_2739 | 57261 | 4.6 | *Ketogulonicigenium vulgare* | 357 |
| alpha/beta hydrolase fold family protein | gkv_2724 | 31756 | 5.48 | *Ketogulonicigenium vulgare* | 222 |
| alpha/beta hydrolase fold family protein | gkv_2723 | 32116 | 5.72 | *Ketogulonicigenium vulgare* | 244 |
| metallopeptidase family M24 family protein | gkv_2722 | 42224 | 5.53 | *Ketogulonicigenium vulgare* | 213 |
| GTP-binding protein TypA/BipA | gkv_2709 | 66421 | 5.12 | *Ketogulonicigenium vulgare* | 85 |
| feS assembly ATPase SufC | gkv_2699 | 27291 | 5.06 | *Ketogulonicigenium vulgare* | 160 |
| ABC transporter, periplasmic substrate-binding protein | gkv_2657 | 36026 | 4.5 | *Ketogulonicigenium vulgare* | 264 |
| ABC transporter, periplasmic substrate-binding protein | gkv_2657 | 36026 | 4.5 | *Ketogulonicigenium vulgare* | 138 |
| translation elongation factor Ts | gkv_2656 | 31828 | 4.91 | *Ketogulonicigenium vulgare* | 218 |
| ribosomal protein S2 | gkv_2655 | 28554 | 5.11 | *Ketogulonicigenium vulgare* | 181 |
| alanine racemase | gkv_2635 | 36811 | 4.87 | *Ketogulonicigenium vulgare* | 138 |
| aconitate hydratase 1 | gkv_2616 | 99849 | 5.02 | *Ketogulonicigenium vulgare* | 143 |
| seryl-tRNA synthetase | gkv_2607 | 47090 | 5.44 | *Ketogulonicigenium vulgare* | 35 |
| seryl-tRNA synthetase | gkv_2607 | 47090 | 5.44 | *Ketogulonicigenium vulgare* | 42 |
| superoxide dismutase [Fe] | gkv_2598 | 22251 | 5.21 | *Ketogulonicigenium vulgare* | 172 |
| activator of Hsp90 ATPase homolog 1-like family protein | gkv_2530 | 17890 | 5.13 | *Ketogulonicigenium vulgare* | 122 |
| succinate-semialdehyde dehydrogenase [NADP+] (SSDH) | gkv_2529 | 50685 | 5.26 | *Ketogulonicigenium vulgare* | 70 |
| ribonuclease D | gkv_2525 | 42832 | 5.51 | *Ketogulonicigenium vulgare* | 224 |
| malate dehydrogenase, NAD-dependent | gkv_246 | 33448 | 5.07 | *Ketogulonicigenium vulgare* | 370 |
| 5'/3'-nucleotidase SurE | gkv_2437 | 窗体顶端  28280  窗体底端 | 5.57 | *Ketogulonicigenium vulgare* | 窗体顶端   304     窗体底端 |
| 5'/3'-nucleotidase SurE | gkv_2437 | 窗体顶端  28280   窗体底端 | 5.57 | *Ketogulonicigenium vulgare* | 217 |
| tat (twin-arginine translocation) pathway signal sequence domain protein | gkv_2425 | 40235 | 4.94 | *Ketogulonicigenium vulgare* | 271 |
| acetolactate synthase, large subunit, biosynthetic type | gkv_2422 | 63505 | 5.73 | *Ketogulonicigenium vulgare* | 82 |
| acetolactate synthase, small subunit | gkv_2421 | 19972 | 5.95 | *Ketogulonicigenium vulgare* | 205 |
| acetolactate synthase, small subunit | gkv_2421 | 19972 | 5.95 | *Ketogulonicigenium vulgare* | 128 |
| cyclophilin type peptidyl-prolyl cis-trans isomerase/CLD family protein | gkv_2418 | 19334 | 4.74 | *Ketogulonicigenium vulgare* | 87 |
| cyclophilin type peptidyl-prolyl cis-trans isomerase/CLD family protein | gkv_2417 | 18529 | 5.48 | *Ketogulonicigenium vulgare* | 103 |
| 3-oxoacyl-(acyl-carrier-protein) reductase | gkv_2387 | 25006 | 5.9 | *Ketogulonicigenium vulgare* | 37 |
| ribosomal protein L9 | gkv_2382 | 20314 | 4.73 | *Ketogulonicigenium vulgare* | 117 |
| trigger factor | gkv_2380 | 48583 | 4.68 | *Ketogulonicigenium vulgare* | 127 |
| enoyl-[acyl-carrier-protein] reductase [NADH] 1 (NADH-dependent enoyl-ACP reductase 1) | gkv_2358 | 29392 | 5.23 | *Ketogulonicigenium vulgare* | 95 |
| xanthine phosphoribosyltransferase (Xanthine-guaninephosphoribosyltransferase) (XGPRT) | gkv_2357 | 19665 | 5.67 | *Ketogulonicigenium vulgare* | 65 |
| phosphoribosylformylglycinamidine synthase I | gkv_2342 | 23669 | 5.76 | *Ketogulonicigenium vulgare* | 69 |
| single-stranded DNA-binding protein (SSB) (Helix-destabilizingprotein) | gkv_2334 | 18661 | 5.92 | *Ketogulonicigenium vulgare* | 326 |
| cysteinyl-tRNA synthetase | gkv_2321 | 49174 | 5.22 | *Ketogulonicigenium vulgare* | 122 |
| basic membrane family protein | gkv_2312 | 35303 | 4.38 | *Ketogulonicigenium vulgare* | 209 |
| membrane-bound lytic murein transglycosylase B-like protein | gkv_2273 | 28523 | 6.9 | *Ketogulonicigenium vulgare* | 89 |
| bacterioferritin | gkv_2223 | 18658 | 4.82 | *Ketogulonicigenium vulgare* | 61 |
| asnC family protein | gkv_2208 | 16419 | 6.15 | *Ketogulonicigenium vulgare* | 145 |
| chaperonin GroL | gkv_2196 | 57419 | 4.97 | *Ketogulonicigenium vulgare* | 293 |
| chaperonin GroL | gkv_2196 | 57419 | 4.97 | *Ketogulonicigenium vulgare* | 271 |
| chaperonin GroL | gkv_2196 | 57419 | 4.97 | *Ketogulonicigenium vulgare* | 110 |
| chaperonin GroL | gkv_2196 | 57419 | 4.97 | *Ketogulonicigenium vulgare* | 70 |
| 10 kDa chaperonin 1 (Protein Cpn10 1) (groES protein 1) | gkv_2195 | 10208 | 5.2 | *Ketogulonicigenium vulgare* | 205 |
| ATP synthase F1, epsilon subunit | gkv_2187 | 12830 | 4.4 | *Ketogulonicigenium vulgare* | 67 |
| ATP synthase F1, beta subunit | gkv_2186 | 50174 | 4.91 | *Ketogulonicigenium vulgare* | 378 |
| ATP synthase F1, beta subunit | gkv_2186 | 50174 | 4.91 | *Ketogulonicigenium vulgare* | 331 |
| ATP synthase F1, beta subunit | gkv_2186 | 50174 | 4.91 | *Ketogulonicigenium vulgare* | 50 |
| ATP synthase F1, alpha subunit | gkv_2184 | 55383 | 5.89 | *Ketogulonicigenium vulgare* | 254 |
| coproporphyrinogen III oxidase family protein | gkv_2169 | 32360 | 5.47 | *Ketogulonicigenium vulgare* | 51 |
| aminotransferase class-III family protein | gkv_2145 | 49867 | 5.26 | *Ketogulonicigenium vulgare* | 292 |
| tryptophanyl-tRNA synthetase | gkv_2112 | 38379 | 5.62 | *Ketogulonicigenium vulgare* | 130 |
| GHMP kinases C terminal family protein | gkv_2067 | 36200 | 5.74 | *Ketogulonicigenium vulgare* | 73 |
| adenine phosphoribosyltransferase | gkv_2039 | 19221 | 5.49 | *Ketogulonicigenium vulgare* | 80 |
| methionine adenosyltransferase | gkv_2011 | 42557 | 5.16 | *Ketogulonicigenium vulgare* | 312 |
| membrane-bound aldehyde dehydrogenase [pyrroloquinoline-quinone] (ALDH) | gkv_201 | 82851 | 4.7 | *Ketogulonicigenium vulgare* | 164 |
| phoH-like family protein | gkv_2007 | 38326 | 5.58 | *Ketogulonicigenium vulgare* | 228 |
| ribosomal protein S1 | gkv_1988 | 窗体顶端  61823 窗体底端 | 窗体顶端  5.02 窗体底端 | *Ketogulonicigenium vulgare* | 62 |
| ribosomal protein S1 | gkv_1987 | 61823 | 5.02 | *Ketogulonicigenium vulgare* | 162 |
| ribosomal protein S1 | gkv_1987 | 61823 | 5.02 | *Ketogulonicigenium vulgare* | 145 |
| putative thiosulfate sulfurtransferase (Rhodanese-likeprotein) | gkv_197 | 32342 | 4.51 | *Ketogulonicigenium vulgare* | 505 |
| putative thiosulfate sulfurtransferase (Rhodanese-likeprotein) | gkv_197 | 32342 | 4.51 | *Ketogulonicigenium vulgare* | 296 |
| aminotransferase class I and II family protein | gkv_1890 | 窗体顶端  42952 窗体底端 | 5.43 | *Ketogulonicigenium vulgare* | 43 |
| transcription elongation factor greA (Transcript cleavage factorgreA) | gkv_1841 | 17121 | 4.83 | *Ketogulonicigenium vulgare* | 73 |
| aldo/keto reductase family protein | gkv_1807 | 33811 | 5.01 | *Ketogulonicigenium vulgare* | 293 |
| aldo/keto reductase family protein | gkv_1807 | 33811 | 5.01 | *Ketogulonicigenium vulgare* | 48 |
| uncharacterized protein in PQQ-III 3'region (ORF R) (Fragment) | gkv_1780 | 37750 | 5.41 | *Ketogulonicigenium vulgare* | 42 |
| nitrogen regulatory protein P-II 1 | gkv_1774 | 12123 | 5.45 | *Ketogulonicigenium vulgare* | 143 |
| dihydroxy-acid dehydratase | gkv_1757 | 61974 | 5.58 | *Ketogulonicigenium vulgare* | 41 |
| calcineurin-like phosphoesterase family protein | gkv_1752 | 46913 | 4.78 | *Ketogulonicigenium vulgare* | 38 |
| universal stress family protein | gkv_1737 | 30100 | 5.14 | *Ketogulonicigenium vulgare* | 164 |
| tat (twin-arginine translocation) pathway signal sequence domain protein | gkv_1693 | 77415 | 4.76 | *Ketogulonicigenium vulgare* | 61 |
| glutathione S-transferase, C-terminal domain protein | gkv_1634 | 30782 | 5.8 | *Ketogulonicigenium vulgare* | 76 |
| fumarylacetoacetate (FAA) hydrolase family protein | gkv_1614 | 24726 | 5.67 | *Ketogulonicigenium vulgare* | 208 |
| AGR_L_2804p, nitrilotriacetate monooxygenase component A homolog ytnJ | gkv_1574 | 49190 | 6.02 | *Ketogulonicigenium vulgare* | 78 |
| ATP-dependent chaperone ClpB | gkv_1465 | 95300 | 5.22 | *Ketogulonicigenium vulgare* | 133 |
| ATP-dependent chaperone ClpB | gkv_1465 | 95300 | 5.22 | *Ketogulonicigenium vulgare* | 147 |
| phosphoglycerate dehydrogenase | gkv_1459 | 56305 | 5.33 | *Ketogulonicigenium vulgare* | 216 |
| NAD(P)H:quinone oxidoreductase | gkv_1452 | 28899 | 5.15 | *Ketogulonicigenium vulgare* | 79 |
| glycosyl hydrolase family 3 N terminal domain protein | gkv_144 | 窗体顶端  37058 窗体底端 | 4.92 | *Ketogulonicigenium vulgare* | 41 |
| S1 RNA binding domain protein | gkv_1433 | 76942 | 5.17 | *Ketogulonicigenium vulgare* | 272 |
| S1 RNA binding domain protein | gkv_1433 | 76942 | 5.17 | *Ketogulonicigenium vulgare* | 78 |
| dihydrodipicolinate reductase | gkv_1427 | 27897 | 5.9 | *Ketogulonicigenium vulgare* | 67 |
| chaperone protein DnaK | gkv_1426 | 68575 | 4.79 | *Ketogulonicigenium vulgare* | 273 |
| chaperone protein DnaK | gkv_1426 | 68575 | 4.79 | *Ketogulonicigenium vulgare* | 342 |
| chaperone protein DnaK | gkv_1426 | 68575 | 4.79 | *Ketogulonicigenium vulgare* | 287 |
| chorismate synthase | gkv_1420 | 39749 | 6.08 | *Ketogulonicigenium vulgare* | 77 |
| response regulator | gkv_1410 | 25875 | 5.37 | *Ketogulonicigenium vulgare* | 96 |
| modulator of DNA gyrase family protein | gkv_1397 | 47926 | 5.06 | *Ketogulonicigenium vulgare* | 73 |
| branched-chain amino acid aminotransferase | gkv_1369 | 32360 | 5.75 | *Ketogulonicigenium vulgare* | 70 |
| inosine-uridine preferring nucleoside hydrolase family protein | gkv_1355 | 34551 | 5.36 | *Ketogulonicigenium vulgare* | 117 |
| protein-export chaperone SecB | gkv_1348 | 19345 | 5.26 | *Ketogulonicigenium vulgare* | 76 |
| grpE family protein | gkv_1333 | 20383 | 4.59 | *Ketogulonicigenium vulgare* | 69 |
| cobalamin-independent synthase, Catalytic domain protein | gkv_1303 | 41881 | 5.59 | *Ketogulonicigenium vulgare* | 290 |
| cobalamin-independent synthase, Catalytic domain protein | gkv_1303 | 41881 | 5.59 | *Ketogulonicigenium vulgare* | 265 |
| cobalamin-independent synthase, Catalytic domain protein | gkv_1303 | 41881 | 5.59 | *Ketogulonicigenium vulgare* | 59 |
| bacterial extracellular solute-binding protein, family 7 family protein | gkv_1302 | 34198 | 4.8 | *Ketogulonicigenium vulgare* | 322 |
| preprotein translocase, SecA subunit | gkv_1295 | 102451 | 5.34 | *Ketogulonicigenium vulgare* | 117 |
| 3-deoxy-D-manno-octulosonate cytidylyltransferase | gkv_1289 | 28994 | 5.05 | *Ketogulonicigenium vulgare* | 88 |
| UTP--glucose-1-phosphate uridylyltransferase | gkv_1288 | 35331 | 6.06 | *Ketogulonicigenium vulgare* | 254 |
| phosphoenolpyruvate-dependent sugar phosphotransferase system, EIIA 2 family protein | gkv_1279 | 16601 | 5.66 | *Ketogulonicigenium vulgare* | 106 |
| nitrilotriacetate monooxygenase component A (NTAmonooxygenase component A) (NTA-MO A) | gkv_1264 | 46750 | 5.68 | *Ketogulonicigenium vulgare* | 116 |
| dTDP-glucose 4,6-dehydratase | gkv_1258 | 38405 | 5.5 | *Ketogulonicigenium vulgare* | 80 |
| aminopeptidase 2 (Aminopeptidase II) (AP-II) | gkv_1237 | 44018 | 5.31 | *Ketogulonicigenium vulgare* | 75 |
| magnesium chelatase, subunit ChlI family protein | gkv_1230 | 35289 | 5.78 | *Ketogulonicigenium vulgare* | 155 |
| transcription termination/antitermination factor NusG | gkv_1199 | 19995 | 5.57 | *Ketogulonicigenium vulgare* | 141 |
| ribosomal protein L11 | gkv_1198 | 15864 | 9.85 | *Ketogulonicigenium vulgare* | 79 |
| ribosomal protein L7/L12 | gkv_1195 | 12773 | 4.86 | *Ketogulonicigenium vulgare* | 134 |
| translation elongation factor G | gkv_1188 | 77895 | 4.98 | *Ketogulonicigenium vulgare* | 240 |
| translation elongation factor G | gkv_1188 | 77895 | 4.98 | *Ketogulonicigenium vulgare* | 136 |
| translation elongation factor G | gkv_1188 | 77895 | 4.98 | *Ketogulonicigenium vulgare* | 98 |
| translation elongation factor Tu | gkv_1187 | 43299 | 5.28 | *Ketogulonicigenium vulgare* | 268 |
| translation elongation factor Tu | gkv_1187 | 43299 | 5.28 | *Ketogulonicigenium vulgare* | 235 |
| 4-deoxy-L-threo-5-hexosulose-uronate ketol-isomerase (5-keto-4-deoxyuronate isomerase) (DKI isomerase) | gkv_118 | 30900 | 6.03 | *Ketogulonicigenium vulgare* | 215 |
| 2-deoxy-D-gluconate 3-dehydrogenase | gkv_117 | 26009 | 6.11 | *Ketogulonicigenium vulgare* | 297 |
| DNA-directed RNA polymerase, alpha subunit | gkv_1159 | 37137 | 4.69 | *Ketogulonicigenium vulgare* | 101 |
| DNA-directed RNA polymerase, alpha subunit | gkv_1159 | 37137 | 4.69 | *Ketogulonicigenium vulgare* | 77 |
| DNA-directed RNA polymerase, alpha subunit | gkv_1159 | 37137 | 4.69 | *Ketogulonicigenium vulgare* | 57 |
| bacterial extracellular solute-binding proteins, family 3 family protein | gkv_113 | 28452 | 4.61 | *Ketogulonicigenium vulgare* | 142 |
| bifunctional enzyme nodQ | gkv_1071 | 69638 | 5.31 | *Ketogulonicigenium vulgare* | 189 |
| homoserine dehydrogenase (HDH) | gkv_1054 | 44588 | 5.95 | *Ketogulonicigenium vulgare* | 198 |
| glutamyl-tRNA(Gln) and/or aspartyl-tRNA(Asn) amidotransferase, A subunit | gkv_1033 | 51898 | 5.06 | *Ketogulonicigenium vulgare* | 224 |
| glutamate synthase [NADPH] small chain (Glutamate synthase subunit beta) (NADPH-GOGAT) (GLTS beta chain) | gkv_1026 | 52394 | 4.9 | *Ketogulonicigenium vulgare* | 76 |
| enolase | gi|56964781 | 46226 | 4.66 | *Bacillus clausii* KSM-K16 | 209 |
| enolase | gi|56964781 | 46226 | 4.66 | *Bacillus clausii* KSM-K16 | 172 |
| enolase | gi|56964781 | 46226 | 4.66 | *Bacillus clausii* KSM-K16 | 73 |
| molecular chaperone DnaK | gi|52786474 | 65909 | 4.78 | *Bacillus licheniformis* ATCC 14580 | 85 |
| pyruvate dehydrogenase (E1 beta subunit) | gi|52080060 | 35482 | 4.8 | *Bacillus licheniformis* ATCC 14580 | 52 |
| aspartate-semialdehyde dehydrogenase | gi|49478383 | 38234 | 5.35 | *Bacillus thuringiensis* serovar konkukian str. 97-27 | 72 |
| aspartate-semialdehyde dehydrogenase | gi|49478383 | 38234 | 5.35 | *Bacillus thuringiensis* serovar konkukian str. 97-27 | 52 |
| asparagine synthetase, glutamine-hydrolyzing | gi|42780333 | 71435 | 6.01 | *Bacillus cereus* ATCC 10987 | 41 |
| KdgR1 protein | gi|311070846 | 40486 | 5.89 | *Ketogulonicigenium vulgare* | 117 |
| isocitrate dehydrogenase | gi|311069409 | 46620 | 4.98 | *Bacillus atrophaeus* 1942 | 87 |
| isocitrate dehydrogenase | gi|311069409 | 46620 | 4.98 | *Bacillus atrophaeus* 1942 | 104 |
| YsdC protein | gi|311069377 | 39357 | 5.73 | *Bacillus atrophaeus* 1942 | 78 |
| YsdC protein | gi|311069377 | 39357 | 5.73 | *Bacillus atrophaeus* 1942 | 103 |
| YsdC protein | gi|311069377 | 39357 | 5.73 | *Bacillus atrophaeus* 1942 | 81 |
| pyridine nucleotide-disulphide oxidoreductase | gi|30265112 | 窗体顶端  41581    窗体底端 | 6.89 | *窗体顶端*  *Bacillus cereus* ATCC 14579 窗体底端 | 窗体顶端   45      窗体底端 |
| glucose-1-dehydrogenase | gi|30264786 | 28044 | 5.8 | *Bacillus anthracis* str. *Ames* | 50 |
| glutamine synthetase, type I | gi|30263707 | 窗体顶端  50531  窗体底端 | 5.04 | *窗体顶端*  *Bacillus subtilis subsp. subtilis* str. 168 窗体底端 | 窗体顶端  50 窗体底端 |
| catalase | gi|30263101 | 62416 | 5.99 | *Bacillus anthracis* str. Ames | 39 |
| 6-phosphogluconate dehydrogenase | gi|30260352 | 52194 | 5.18 | *Bacillus anthracis* str. Ames | 71 |
| Thiol peroxidase | gi|30022714 | 窗体顶端  18033 窗体底端 | 窗体顶端  4.85 窗体底端 | *Ketogulonicigenium vulgare* | 113 |
| glycine dehydrogenase subunit 2 | gi|30022307 | 59712 | 5.43 | *Bacillus cereus* ATCC 14579 | 36 |
| glycine dehydrogenase subunit 2 | gi|30022307 | 59712 | 5.43 | *Bacillus cereus* ATCC 14579 | 36 |
| succinyl-CoA synthetase subunit beta | gi|30021924 | 41954 | 5 | *Bacillus cereus* ATCC 14579 | 324 |
| Serine protein kinase | gi|30018736 | 73282 | 5.75 | *Bacillus cereus* ATCC 14579 | 58 |
| inositol-5-monophosphate dehydrogenase | gi|30018286 | 52652 | 6.45 | *Bacillus cereus* ATCC 14579 | 84 |
| chaperonin GroEL | gi|297582890 | 57297 | 4.58 | *Bacillus selenitireducens* MLS10 | 117 |
| glutamyl-tRNA(Gln) amidotransferase, A subunit | gi|295697351 | 52840 | 5.36 | *Bacillus tusciae* DSM 2912 | 65 |
| catalase | gi|294501951 | 54874 | 5.92 | *Bacillus megaterium* QM B1551 | 78 |
| catalase | gi|294501951 | 54874 | 5.92 | *Bacillus megaterium* QM B1551 | 78 |
| catalase | gi|294501951 | 54874 | 5.92 | *Bacillus megaterium* QM B1551 | 42 |
| succinate dehydrogenase, flavoprotein subunit | gi|294501460 | 66543 | 5.98 | *Bacillus megaterium* QM B155 | 92 |
| chorismate mutase/phospho-2-dehydro-3-deoxyheptonate aldolase | gi|294499757 | 39375 | 5.66 | *Bacillus megaterium* QM B1551 | 112 |
| xylose isomerase domain protein TIM barrel | gi|294498913 | 36615 | 5 | *Bacillus megaterium* QM B1551 | 135 |
| delta-1-pyrroline-5-carboxylate dehydrogenase | gi|294497429 | 56842 | 5.36 | *Bacillus megaterium* QM B1551 | 115 |
| septation protein SpoVG | gi|294496920 | 10701 | 5.11 | *Bacillus megaterium* QM B1551 | 113 |
| hypothetical protein BpOF4_19550 | gi|288556906 | 29126 | 6.18 | *Bacillus pseudofirmus* OF4 | 36 |
| superoxide dismutase | gi|288555787 | 22452 | 5.18 | *Bacillus pseudofirmus* OF4 | 159 |
| superoxide dismutase | gi|288555787 | 22452 | 5.18 | *Bacillus pseudofirmus* OF4 | 119 |
| adenylosuccinate synthetase | gi|288554582 | 47734 | 5.38 | *Bacillus pseudofirmus* OF4 | 49 |
| putative dehydrogenase | gi|16081077 | 43376 | 5.82 | *Bacillus subtilis* subsp. subtilis str. 168 | 58 |
| 1-pyrroline-5-carboxylate dehydrogenase | gi|16080830 | 56513 | 5.67 | *Bacillus subtilis* subsp. subtilis str. 168 | 139 |
| fructose-bisphosphate aldolase | gi|16080765 | 30552 | 5.19 | *Bacillus subtilis* subsp. subtilis str. 168 | 59 |
| glyceraldehyde-3-phosphate dehydrogenase | gi|16080447 | 35924 | 5.2 | *Bacillus subtilis* subsp. subtilis str. 168 | 68 |
| glyceraldehyde-3-phosphate dehydrogenase | gi|16080447 | 35924 | 5.2 | *Bacillus subtilis* subsp. subtilis str. 168 | 107 |
| glyceraldehyde-3-phosphate dehydrogenase | gi|16080447 | 35924 | 5.2 | *Bacillus subtilis* subsp. subtilis str. 168 | 67 |
| glyceraldehyde-3-phosphate dehydrogenase | gi|16080447 | 35924 | 5.2 | *Bacillus subtilis* subsp. subtilis str. 168 | 121 |
| malate dehydrogenase | gi|16079964 | 33623 | 4.92 | *Bacillus subtilis* subsp. subtilis str. 168 | 315 |
| molecular chaperone DnaK | gi|16079601 | 66019 | 4.76 | *Bacillus subtilis* subsp. subtilis str. 168 | 114 |
| peptidyl-prolyl isomerase | gi|16079393 | 15360 | 5.53 | *Bacillus subtilis* subsp. subtilis str. 168 | 100 |
| morphogenetic stage IV sporulation protein | gi|16079337 | 55197 | 4.74 | *Bacillus subtilis* subsp. subtilis str. 168 | 67 |
| nucleoside diphosphate kinase | gi|16079330 | 16948 | 5.67 | *Bacillus subtilis* subsp. subtilis str. 168 | 57 |
| serine protein kinase | gi|16077962 | 73013 | 5.58 | *Bacillus subtilis* subsp. subtilis str. 168 | 39 |
| DNA-directed RNA polymerase subunit alpha | gi|16077211 | 34835 | 4.8 | *Bacillus subtilis* subsp. subtilis str. 168 | 52 |
| DNA-directed RNA polymerase subunit alpha | gi|16077211 | 34835 | 4.8 | *Bacillus subtilis* subsp. subtilis str. 168 | 58 |
| cysteine synthase | gi|16077141 | 32799 | 5.64 | *Bacillus subtilis* subsp. subtilis str. 168 | 104 |
| putative manganese-dependent inorganic pyrophosphatase | gi|157694450 | 33702 | 4.64 | *Bacillus pumilus* SAFR-032 | 60 |
| succinate dehydrogenase flavoprotein subunit | gi|157693262 | 65152 | 5.59 | *Bacillus pumilus* SAFR-032 | 151 |
| methylmalonate-semialdehyde dehydrogenase (acylating) | gi|157692519 | 53459 | 5.29 | *Bacillus pumilus* SAFR-032 | 80 |
| putative translaldolase | gi|15616347 | 22967 | 5.77 | *Bacillus halodurans* C-125 | 40 |
| F0F1 ATP synthase subunit alpha | gi|15616318 | 54669 | 5.22 | *Bacillus halodurans* C-125 | 190 |
| F0F1 ATP synthase subunit alpha | gi|15616318 | 54669 | 5.22 | *Bacillus halodurans* C-125 | 188 |
| F0F1 ATP synthase subunit alpha | gi|15616318 | 54669 | 5.22 | *Bacillus halodurans* C-125 | 177 |
| F0F1 ATP synthase subunit alpha | gi|15616318 | 54669 | 5.22 | *Bacillus halodurans* C-125 | 148 |
| ATP-dependent Clp protease proteolytic subunit | gi|15616126 | 21505 | 5.48 | *Bacillus halodurans* C-125 | 107 |
| thioredoxin peroxidase | gi|15615756 | 18033 | 4.85 | *Bacillus halodurans* C-125 | 119 |
| pyruvate kinase | gi|15615725 | 62412 | 5.06 | *Bacillus halodurans* C-125 | 115 |
| alanine dehydrogenase (stage V sporulation protein N) | gi|15614892 | 39716 | 5.1 | *Bacillus halodurans* C-125 | 80 |
| acetoin dehydrogenase E1 component (TPP-dependent alpha subunit) | gi|15614385 | 37320 | 5.15 | *Bacillus halodurans* C-125 | 52 |
| stage IV sporulation protein A (spore cortex formation and coat assembly) | gi|15614208 | 55553 | 4.75 | *Bacillus halodurans* C-125 | 36 |
| 6,7-dimethyl-8-ribityllumazine synthase | gi|15614120 | 16399 | 5.39 | *Bacillus halodurans* C-125 | 109 |
| chaperonin GroEL | gi|15613125 | 57368 | 4.79 | *Bacillus halodurans* C-125 | 49 |
| elongation factor Tu | gi|15612695 | 43470 | 4.79 | *Bacillus halodurans* C-125 | 335 |
| elongation factor Tu | gi|15612695 | 43470 | 4.79 | *Bacillus halodurans* C-125 | 329 |
| elongation factor Tu | gi|15612695 | 43470 | 4.79 | *Bacillus halodurans* C-125 | 38 |
| elongation factor Tu | gi|15612695 | 43470 | 4.79 | *Bacillus halodurans* C-125 | 43 |
| seryl-tRNA synthetase | gi|15612587 | 48625 | 5.51 | *Bacillus halodurans* C-125 | 63 |
| elongation factor G | gi|154684630 | 76601 | 4.8 | *Bacillus amyloliquefaciens* FZB42 | 95 |
| stress protein | gi|152974220 | 21185 | 4.65 | *Bacillus cereus* subsp. cytotoxis NVH 391-98 | 115 |
| stress protein | gi|152974220 | 21185 | 4.65 | *Bacillus cereus* subsp. cytotoxis NVH 391-98 | 129 |
